# Efficacy of Different Combination Therapies for Mania in Bipolar Disorder: A Systematic Review and Meta‐Analysis

**DOI:** 10.1002/brb3.71139

**Published:** 2025-12-22

**Authors:** Peiling Yao, Pingan Ni, Liqin Yin

**Affiliations:** ^1^ Department of Psychosomatic Disorders Huzhou Third People's Hospital Huzhou Zhejiang China

**Keywords:** bipolar disorder, manic episode, meta‐analysis, mood stabilizers, olanzapine, ziprasidone

## Abstract

**Objective:**

To systematically evaluate the efficacy and safety of different combination therapies in the treatment of mania in bipolar disorder, and to provide evidence‐based support for individualized clinical treatment.

**Methods:**

PubMed, Web of Science, Cochrane Library, CNKI, VIP, and Wanfang databases were systematically searched from inception to July 2025. Randomized controlled trials comparing ziprasidone or olanzapine combined with mood stabilizers (lithium carbonate or sodium valproate) for the treatment of manic episodes in bipolar disorder were included. Two reviewers independently performed literature screening, data extraction, and quality assessment. Meta‐analysis was conducted using RevMan 5.4 software.

**Results:**

A total of 10 RCTs involving 842 patients were included. Meta‐analysis showed no significant difference between the two groups in overall clinical efficacy (OR = 1.18, 95% CI: 0.80–1.75, *p* = 0.41). Ziprasidone was superior to olanzapine in reducing YMRS scores (MD = −1.47, 95% CI: −1.97–0.96, *p* < 0.00001). The incidence of adverse reactions was significantly lower in the ziprasidone group compared to the olanzapine group (OR = 0.16, 95% CI: 0.08–0.31, *p* < 0.00001). Furthermore, ziprasidone was associated with significantly greater increases in BDNF and T_3_ levels after treatment.

**Conclusion:**

Although the efficacy of ziprasidone plus mood stabilizers is comparable to that of olanzapine plus mood stabilizers in the treatment of manic episodes of bipolar disorder, ziprasidone offers advantages in improving manic symptoms (YMRS scores), reducing adverse events, and enhancing neuroendocrine indicators. It may serve as a favorable alternative in clinical practice. Further high‐quality, multicenter, large‐sample studies are needed to confirm its efficacy and safety.

## Graphical abstract

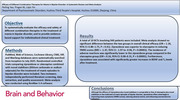

## 1 Introduction

Bipolar disorder (BD) is a chronic mental illness characterized by alternating or mixed episodes of elevated mood (mania or hypomania) and depressed mood. It is highly recurrent, associated with significant functional impairment, and carries a high risk of disability and suicide (Raeder et al. 2025; Yang and Liu 2018). The lifetime prevalence worldwide is approximately 1%–2%. However, clinical diagnosis is often delayed or inaccurate, further exacerbating the disease burden (Jing et al. 2009; Cichoń et al. 2020; Xu 2022; Wen and Liu 2014). As a critical phase of BD, manic episodes often present with elevated mood, irritability, racing thoughts, increased activity, impulsive behavior, and, in severe cases, psychotic symptoms (Cichoń et al. 2020; Salazar De Pablo et al. 2020). If mania is not promptly and effectively controlled, patients may experience severe social dysfunction and progress to rapid cycling or depressive episodes, adversely affecting long‐term disease outcomes.

Pharmacotherapy remains the primary approach for managing manic episodes. Current clinical guidelines widely recommend mood stabilizers (such as lithium carbonate and sodium valproate) and second‐generation antipsychotic drugs (SGAs) as first‐line treatments (Kishi et al. 2021). Although mood stabilizers demonstrate clear efficacy in alleviating manic symptoms, their onset of action is relatively slow, and some patients exhibit an inadequate response to monotherapy (Wang et al. 2018; Sun 2021). Therefore, combination therapy with SGAs has become a common strategy in the acute treatment of mania, enhancing therapeutic effects, accelerating symptom relief, and improving remission rates. Among SGAs commonly used in clinical practice, ziprasidone and olanzapine have attracted significant attention and research due to their distinct differences in pharmacological mechanisms, speed of onset, and adverse reaction profiles (Xu 2022; Wen and Liu 2014). Ziprasidone is a multi‐receptor modulating antipsychotic agent that acts as an antagonist at both 5‐HT_2_A and D_2_ receptors, while also inhibiting the reuptake of serotonin and norepinephrine (Stahl and Shayegan 2003; J. Gao, Huang, et al. 2019). It is considered to have potential advantages in improving affective symptoms and is associated with a milder adverse effect profile, particularly with respect to weight gain and metabolic disturbances, compared to other SGAs. As a result, it is widely used in combination therapy during acute manic episodes (Luo et al. 2012; Hu et al. 2019). In contrast, olanzapine demonstrates stable efficacy and rapid onset in the acute treatment of mania, with a long history of clinical use. However, it is associated with significant metabolic side effects, such as weight gain, hyperglycemia, and dyslipidemia, which often lead to poor patient adherence (Citrome et al. 2021; Haddad et al. 2022). There remains some controversy regarding the comparative efficacy and safety of ziprasidone and olanzapine when used in combination with mood stabilizers for treating manic episodes.

Current clinical studies have attempted to compare the therapeutic effects of ziprasidone and olanzapine combined with mood stabilizers in patients with manic episodes of BD. However, limitations such as small sample sizes, variability in intervention protocols, and insufficient follow‐up duration remain. Furthermore, differences in receptor binding profiles, clinical onset characteristics, and metabolic adverse effects between the two drugs pose challenges for clinical decision‐making. These factors have led to inconsistent research conclusions, and a lack of systematic evidence persists (Chen et al. 2014; Y. S. Gao, Chen, et al. 2019). Meta‐analysis can integrate data from multiple randomized controlled trials (RCTs) to enhance statistical power and clarify the comparative advantages of different treatment regimens, thereby providing more reliable evidence for individualized clinical therapy. Therefore, this study aims to systematically evaluate the efficacy and adverse reaction risks of ziprasidone versus olanzapine combined with mood stabilizers in the treatment of manic episodes in BD by synthesizing existing randomized controlled trials through systematic review and meta‐analysis. The findings are expected to offer scientific guidance for clinical practice and establish a foundation for future related research. Given that the currently available direct comparative studies are primarily from China, we will thoroughly discuss the impact of regional origin on the generalizability of the results and advocate for future multicenter, multinational, large‐sample studies to further validate and extend our findings.

## 2 Methods

### 2.1 Search Strategy

This study was designed and reported in accordance with the PRISMA (Preferred Reporting Items for Systematic Reviews and Meta‐Analyses) statement (Page et al. 2021). A comprehensive systematic search was conducted across multiple Chinese and English databases to obtain as extensive literature as possible. The databases searched included PubMed, Web of Science, the Cochrane Library, China National Knowledge Infrastructure (CNKI), VIP, and Wanfang Database. The search period spanned from inception to July 2025, with language restrictions limited to Chinese and English. The search strategy incorporated both subject headings and free‐text terms, with the main keywords including: “ziprasidone,” “olanzapine,” “bipolar disorder,” “mania,” “mood stabilizer,” “lithium,” “valproate,” and “randomized controlled trial.” The search logic and expression methods vary slightly across different databases, and adjustments were made according to the actual circumstances. In addition, potential omitted literature was supplemented by manually searching the reference lists of included articles, review literature, and conference abstracts. When necessary, the original authors were contacted to obtain unpublished important data.

### 2.2 Inclusion and Exclusion Criteria

Studies were included only if they met all of the following five criteria:

1. The study type was an RCT that directly compared the therapeutic effects of ziprasidone combined with mood stabilizers versus olanzapine combined with mood stabilizers;
2. Study participants were inpatients or outpatients diagnosed with the manic episode of BD according to international diagnostic criteria such as DSM or ICD (Fredskild et al. 2021);
3. Intervention: the experimental group received ziprasidone combined with mood stabilizers such as lithium carbonate or sodium valproate, while the control group received olanzapine combined with the same type of mood stabilizer;
4. The study reported at least one of the following primary clinical outcome measures: overall clinical response rate, Young Mania Rating Scale (YMRS), incidence of adverse reactions, brain‐derived neurotrophic factor (BDNF) level, and triiodothyronine (T_3_) level;
5. The study data were complete, allowing extraction of statistical data such as effective sample size and mean ± standard deviation.

Exclusion criteria comprised: interventions that did not explicitly combine mood stabilizers, or where the administered drugs did not align with the control/experimental group design; studies published only as conference abstracts or with incomplete data that precluded statistical meta‐analysis; duplicate publications or studies with overlapping subject populations, in which case only the most recent or most comprehensive version was retained.

### 2.3 Literature Screening and Data Extraction

Literature screening and data extraction were independently performed by two researchers. Initial screening was conducted based on titles and abstracts to exclude studies that clearly did not meet the inclusion criteria. The full texts of the remaining articles were then reviewed to determine final eligibility according to the predefined inclusion and exclusion criteria. Data extracted included basic study information (first author, publication year), sample size and patient baseline characteristics (mean age, gender, disease duration), intervention protocols, primary efficacy outcomes (e.g., YMRS score, overall response rate), and safety indicators (e.g., incidence of adverse events). All data were extracted separately by two investigators and cross‐checked. Discrepancies or missing data were resolved by contacting the original authors for raw data or through adjudication by a third researcher.

### 2.4 Quality Assessment and Risk of Bias Evaluation

The Cochrane Risk of Bias Tool (RoB 2.0) was used to systematically evaluate the methodological quality of the included randomized controlled trials. Key domains assessed included: appropriateness of random sequence generation, allocation concealment, blinding of participants, personnel, and outcome assessors, completeness of outcome data, selective reporting, and other potential sources of bias. Each domain was classified as “low risk,” “high risk,” or “unclear risk”. Two reviewers independently performed the assessments. Disagreements were resolved through discussion or by consultation with a third reviewer. This process helped ensure the overall quality of the included studies and enhanced the rigor and credibility of the conclusions.

### 2.5 Statistical Analysis Methods

Meta‐analysis was conducted using RevMan 5.4 software. Continuous variables (e.g., changes in YMRS scores) were expressed as a mean difference (MD) with 95% confidence interval (CI), while dichotomous variables (e.g., overall response rate, incidence of adverse events) were expressed as odds ratio (OR) with 95% CI. Heterogeneity among studies was assessed using Cochran's *Q* test and the *I^2^* statistic. An *I^2^* value below 50% indicated mild heterogeneity, and a fixed‐effects model was applied; otherwise, a random‐effects model was used. Publication bias was evaluated using Egger's regression test to ensure the robustness and reliability of the findings.

## 3 Results

### 3.1 Literature Screening Process

**FIGURE 1:**
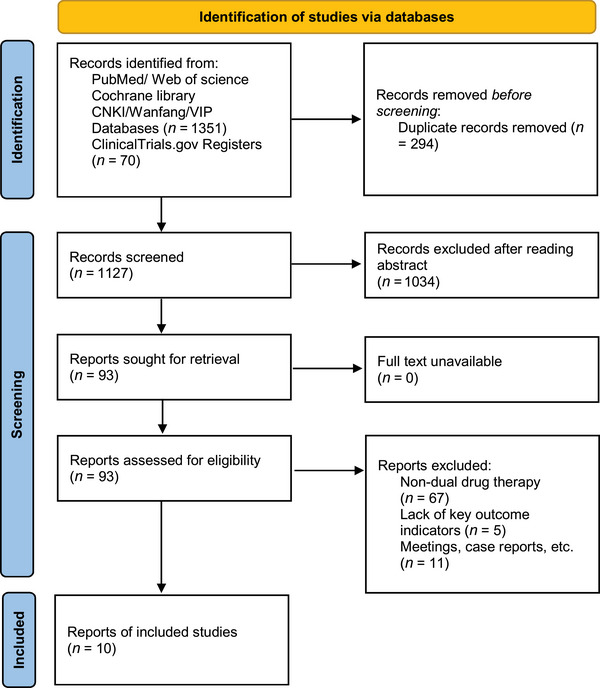
Literature screening flowchart (PRISMA flowchart).

This study initially retrieved a total of 1421 records from databases including PubMed, Web of Science, Cochrane Library, CNKI, VIP, and Wanfang. Supplementary searches for grey literature were conducted via Google Scholar, and relevant registered trials were identified via the ClinicalTrials.gov Registry. After removing 294 duplicates, titles and abstracts were screened, leading to the exclusion of 1034 records that clearly did not meet the inclusion criteria. The remaining 93 full‐text articles were assessed for eligibility. After full‐text review, 67 articles not employing the two‐drug combination therapy regimen, five articles not reporting key outcome indicators, and 11 other records (e.g., conference abstracts, case reports) were excluded. Finally, 10 studies meeting the criteria (Yang and Liu 2018; Xu 2022; Wen and Liu 2014; Wang et al. 2018; Sun 2021; Luo et al. 2012; Hu et al. 2019; Chen et al. 2014; Y. S. Gao, Chen, et al. 2019; Chen 2021) were included for the subsequent meta‐analysis (Figure 1). Furthermore, the supplementary search via Google Scholar did not yield any additional eligible studies. These results are incorporated into the PRISMA flow diagram.

### 3.2 Basic Characteristics and Quality Assessment

A total of 10 randomized controlled trials (Yang and Liu 2018; Xu 2022; Wen and Liu 2014; Wang et al. 2018; Sun 2021; Luo et al. 2012; Hu et al. 2019; Chen et al. 2014; Y. S. Gao, Chen, et al. 2019; Chen 2021) were included in this study, comprising a total sample size of 842 participants, with roughly equal numbers in the experimental and control groups. All participants were diagnosed with BD during the manic phase. The interventions involved ziprasidone or olanzapine combined with mood stabilizers (lithium carbonate or sodium valproate), with treatment durations ranging from 4 to 12 weeks, as detailed in Table 1. Quality assessment results indicated that all studies clearly employed random allocation, with a low risk of bias in random sequence generation. However, most studies did not report allocation concealment or blinding procedures, presenting potential risks of selection and detection bias. All studies had complete outcome data, with no omissions in primary outcomes, and exhibited a low risk of selective reporting and other biases. Overall, the methodological quality of the included studies was moderate to high, as detailed in Table 2.

**TABLE 1:** General characteristics of included studies.

Author	Year of publication	Sample size		Age (years)		Disease duration (months)		Number of episodes		Intervention in experimental group	Intervention in control group	Treatment duration
		Experimental group	Control group	Experimental group	Control group	Experimental group	Control group	Experimental group	Control group			
(Yang and Liu 2018)	2018	60	60	43.21 ± 5.6	44.04 ± 6.2	4.2 ± 0.4	4.4 ± 0.5	N/A	N/A	Ziprasidone + Lithium Carbonate	Olanzapine + Lithium Carbonate	12 weeks
(Xu 2022)	2022	40	40	42.62 ± 3.58	41.48 ± 3.29	3.71 ± 0.25	3.62 ± 0.59	2.45 ± 0.73	2.13 ± 0.31	Ziprasidone + Sodium Valproate	Olanzapine + Sodium Valproate	6 weeks
(Wen and Liu 2014)	2014	32	32	36.7 ± 6.5	36.7 ± 6.5	N/A	N/A	N/A	N/A	Ziprasidone + Sodium Valproate	Olanzapine + Sodium Valproate	8 weeks
(Wang et al. 2018)	2018	50	50	41.78 ± 17.96	41.05 ± 18.32	N/A	N/A	N/A	N/A	Ziprasidone + Sodium Valproate	Olanzapine + Sodium Valproate	8 weeks
(Sun 2021)	2021	43	43	35.72 ± 6.01	35.67 ± 5.98	1.68 ± 0.49	1.65 ± 0.52	N/A	N/A	Ziprasidone + Sodium Valproate	Olanzapine + Sodium Valproate	8 weeks
(Luo et al. 2012)	2012	24	22	38 ± 13	33 ± 12	N/A	N/A	N/A	N/A	Ziprasidone + Sodium Valproate	Olanzapine + Sodium Valproate	4 weeks
(Hu et al. 2019)	2019	42	42	44.92 ± 5.27	45.61 ± 5.40	4.27 ± 0.82	4.10 ± 0.76	N/A	N/A	Ziprasidone + Sodium Valproate	Olanzapine + Sodium Valproate	8 weeks
(Chen et al. 2014)	2014	50	50	28.5 ± 15.8	30.1 ± 16.6	1.5 ± 1.0	0.2 ± 0.5	1. 5 ± 0.5	1. 3 ± 0.4	Ziprasidone + Lithium Carbonate	Olanzapine + Lithium Carbonate	8 weeks
(Y. S. Gao, Chen, et al. 2019)	2019	49	49	35.51 ± 4.17	36.84 ± 3.98	4.49 ± 0.78	4.35 ± 0.67	1.73 ± 0.25	1.68 ± 0.20	Ziprasidone + Sodium Valproate	Olanzapine + Sodium Valproate	8 weeks
(Chen 2021)	2021	32	32	34.27 ± 3.73	33.64 ± 3.86	N/A	N/A	N/A	N/A	Ziprasidone + Sodium Valproate	Olanzapine + Sodium Valproate	4 weeks

**TABLE 2:** Literature quality assessment.

Study	Random sequence generation	Allocation concealment	Blinding	Completeness of outcomes	Selective reporting	Other biases
(Yang and Liu 2018)	Low risk	Unclear risk	Unclear risk	Low risk	Low risk	Low risk
(Xu 2022)	Low risk	Unclear risk	Unclear risk	Low risk	Low risk	Low risk
(Wen and Liu 2014)	Low risk	Unclear risk	Unclear risk	Low risk	Low risk	Low risk
(Wang et al. 2018)	Low risk	Unclear risk	Unclear risk	Low risk	Low risk	Low risk
(Sun 2021)	Low risk	Unclear risk	Unclear risk	Low risk	Low risk	Low risk
(Luo et al. 2012)	Low risk	Unclear risk	Unclear risk	Low risk	Low risk	Low risk
(Hu et al. 2019)	Low risk	Unclear risk	Unclear risk	Low risk	Low risk	Low risk
(Chen et al. 2014)	Low risk	Unclear risk	High risk	Low risk	Low risk	Low risk
(Y. S. Gao, Chen, et al. 2019)	Low risk	Unclear risk	High risk	Low risk	Low risk	Low risk
(Chen 2021)	Low risk	Unclear risk	Unclear risk	Low risk	Low risk	Low risk

### 3.3 Primary Outcome Analysis

#### 3.3.1 Clinical Effective Rate

**FIGURE 2:**
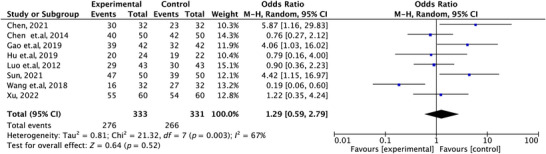
Forest plot of meta‐analysis on clinical response rates between ziprasidone combined with mood stabilizers and olanzapine combined with mood stabilizers.

A total of eight studies (Xu 2022; Wang et al. 2018; Sun 2021; Luo et al. 2012; Hu et al. 2019; Chen et al. 2014; Y. S. Gao, Chen, et al. 2019; Chen 2021) reported the clinical effective rate of ziprasidone combined with mood stabilizers versus olanzapine combined with mood stabilizers in the treatment of manic episodes in BD. Heterogeneity analysis indicated a moderate degree of heterogeneity among the included studies (*I^2^* = 67%), and thus a random‐effects model was applied. The results showed that the number of effective cases was 276 in the experimental group and 266 in the control group. Pooled analysis revealed no statistically significant difference in efficacy between the two groups (OR = 1.29, 95% CI: 0.59–2.79, *p* = 0.52), as shown in Figure 2, indicating that the combination therapy with ziprasidone was comparable to that with olanzapine in terms of clinical effectiveness. Potential sources of heterogeneity may include variations in sample sizes, types of mood stabilizers (lithium or valproate), differences in treatment duration, and methodological factors such as the lack of blinding in some studies.

#### 3.3.2 YMRS

**FIGURE 3:**
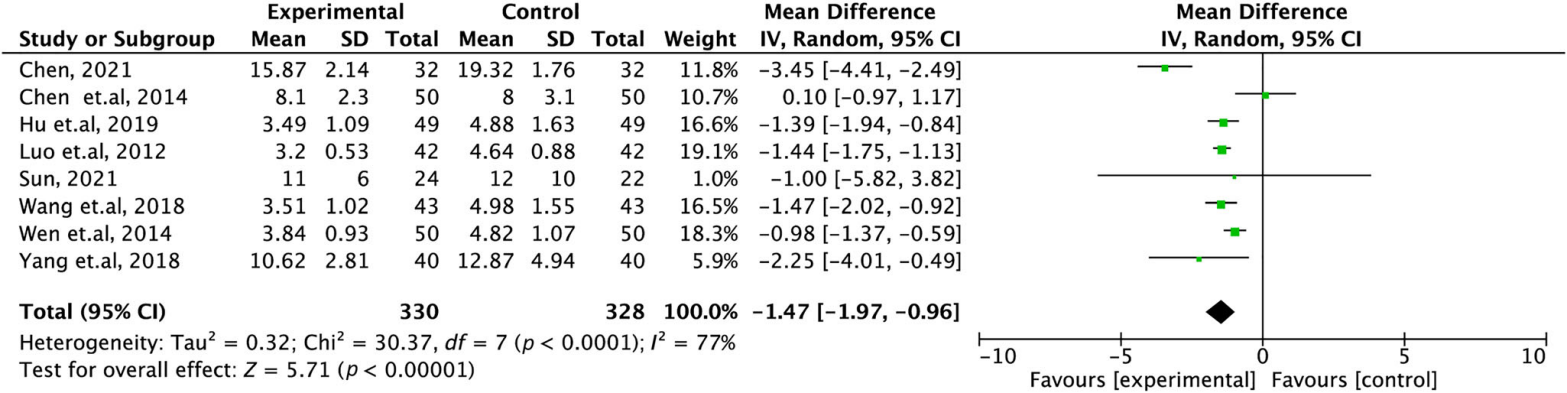
Forest plot of meta‐analysis for YMRS scores after treatment in the ziprasidone combined with mood stabilizers group versus the olanzapine combined with mood stabilizers group.

A total of eight studies (Yang and Liu 2018; Wen and Liu 2014; Wang et al. 2018; Sun 2021; Luo et al. 2012; Hu et al. 2019; Chen et al. 2014; Chen 2021) were included in this analysis comparing YMRS scores before and after treatment. The YMRS score was used as the primary continuous outcome measure. Each study provided the mean and standard deviation of YMRS scores for both the experimental and control groups at the end of treatment. Meta‐analysis results indicated that the experimental group (ziprasidone combined with mood stabilizers) demonstrated superior efficacy in reducing YMRS scores compared to the control group (olanzapine combined with mood stabilizers). The pooled mean difference was MD = −1.47, with a 95% CI: −1.97–0.96. The difference was statistically significant (*p* < 0.00001), as shown in Figure 3, suggesting that ziprasidone combination therapy may offer certain advantages in terms of YMRS improvement. Heterogeneity testing revealed an *I^2^* value of 77%, indicating substantial heterogeneity among the included studies. This may be attributed to variations in the types of mood stabilizers used, treatment durations, enrollment criteria, or baseline YMRS scores across different studies.

#### 3.3.3 Incidence of Adverse Reactions

**FIGURE 4:**
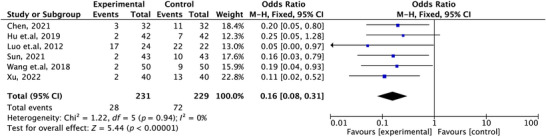
Forest plot of meta‐analysis on the incidence of adverse reactions during treatment between the ziprasidone combined with mood stabilizers group and the olanzapine combined with mood stabilizers group.

A total of six studies (Xu 2022; Wang et al. 2018; Sun 2021; Luo et al. 2012; Hu et al. 2019; Chen 2021) reported the incidence of adverse reactions during treatment. The meta‐analysis results showed that the incidence of adverse reactions in the ziprasidone combined with mood stabilizers group was significantly lower than that in the olanzapine combined with mood stabilizers group, with a pooled OR of 0.16, 95% CI: 0.08–0.31. The difference was highly statistically significant (*p* < 0.00001), as shown in Figure 4. This indicates that, with comparable efficacy, ziprasidone combination therapy offers better safety and tolerability. The heterogeneity test results showed *I^2^* = 0%, indicating low heterogeneity among the studies. Therefore, a fixed‐effects model was used for the pooled analysis, and the results are considered robust.

#### 3.3.4 BDNF

**FIGURE 5:**
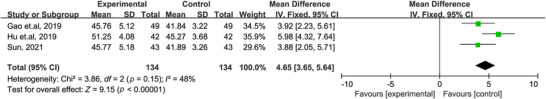
Forest plot of meta‐analysis for BDNF levels (ng/mL) after treatment in the ziprasidone combined with mood stabilizers group versus the olanzapine combined with mood stabilizers group.

A total of three studies (Sun 2021; Hu et al. 2019; Y. S. Gao, Chen, et al. 2019) reported changes in patients' BDNF levels before and after treatment. The meta‐analysis results indicated that the increase in BDNF levels was significantly greater in the ziprasidone combined with mood stabilizers group compared to the olanzapine combination therapy group. The pooled MD was 4.65, with a 95% CI: 3.65–5.64, and the difference was highly statistically significant (*p* < 0.00001), as shown in Figure 5. This suggests that ziprasidone may offer greater advantages in improving neuroplasticity and neurotrophic function. Heterogeneity analysis revealed *I^2^* = 48%, indicating low to moderate heterogeneity; therefore, a fixed‐effects model was used for the pooled analysis.

#### 3.3.5 T_3_

**FIGURE 6:**
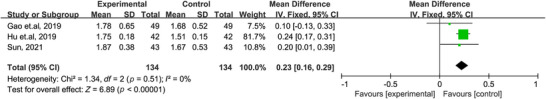
Forest plot of meta‐analysis on serum T_3_ levels (nmol/mL) after treatment in the ziprasidone combined with mood stabilizers group versus the olanzapine combined with mood stabilizers group.

A total of three studies (Sun 2021; Hu et al. 2019; Y. S. Gao, Chen, et al. 2019) reported changes in serum T_3_ levels after treatment. The meta‐analysis results indicated that the increase in T_3_ levels was greater in the ziprasidone combined with mood stabilizers group compared to the olanzapine combined treatment group. The pooled MD was 0.23, with a 95% CI: 0.16–0.29. The difference was statistically significant (*p* < 0.00001), as shown in Figure 6, suggesting that ziprasidone combination therapy may offer greater advantages in regulating thyroid function. Heterogeneity analysis revealed an *I^2^* = 0%, indicating very low heterogeneity among the studies and consistent, stable results. A fixed‐effects model was used for the pooled analysis.

### 3.4 Publication Bias

To assess potential publication bias in the included studies, Egger's regression test was employed to evaluate outcome indicators. The results showed that all *p*‐values were > 0.05, indicating no statistically significant publication bias.

## 4 Discussion

BD is a severe mental disorder characterized by alternating episodes of mania and depression. During manic episodes, patients often present with elevated mood, irritability, racing thoughts, and impulsive behavior. These symptoms not only significantly impair social functioning and interpersonal relationships but also substantially increase the risk of self‐harm, aggression, and hospitalization (Okpaleke Amazu et al. 2024; Turan et al. 2023). Therefore, timely, effective, and safe intervention strategies are crucial for controlling disease progression and improving prognosis. In recent years, with in‐depth research into the disease mechanisms, SGAs combined with mood stabilizers have become first‐line treatments for manic episodes. The goals of such therapy are to achieve rapid symptom remission, reduce relapse rates, and enhance patient adherence and quality of life (Luo et al. 2012; Hu et al. 2019). In this meta‐analysis, we systematically evaluated the efficacy and safety of two commonly used SGAs—ziprasidone and olanzapine—in combination with mood stabilizers for the treatment of manic episodes in BD. The analysis included 10 RCTs involving a total of 842 patients, providing results with considerable representativeness and practical value.

In terms of the primary efficacy outcome, the combination of ziprasidone with mood stabilizers demonstrated comparable clinical overall response rates to olanzapine combination therapy, with no statistically significant difference between the two groups. This indicates that both regimens are effective in controlling core manic symptoms. The overall therapeutic effect of such combination strategies aligns with current clinical recommendations in multiple international guidelines and reflects the synergistic benefits of combining SGAs with mood stabilizers, particularly in moderate to severe manic episodes (Wang et al. 2018; Sun 2021; Luo et al. 2012). Further analysis of changes in YMRS scores, however, revealed that ziprasidone provided greater improvement in manic symptom severity compared to olanzapine. This finding suggests that ziprasidone may offer advantages in either the speed or depth of symptom reduction, warranting further investigation and validation during acute‐phase intervention. The difference may stem from their distinct pharmacological mechanisms. Olanzapine exerts its robust antimanic effects primarily through potent antagonism of dopamine D_2_ and serotonin 5‐HT_2_A receptors. However, its high affinity for histaminergic and cholinergic receptors contributes to notable side effects such as sedation, weight gain, and metabolic disturbances (Zhou et al. 2022; Tiwari et al. 2018). In contrast, ziprasidone not only acts as an antagonist at D_2_ and 5‐HT_2_A receptors but also exhibits partial agonism at 5‐HT_1_A receptors and inhibits serotonin and norepinephrine reuptake. This multimodal activity may contribute to its broader potential in regulating mood (J. Gao, Huang, et al. 2019; Fredskild et al. 2021). Moreover, ziprasidone has lower affinity for muscarinic and H_1_ histaminergic receptors, resulting in a more favorable metabolic side effect profile and less sedation, thereby better supporting patients' daytime functioning.

This meta‐analysis demonstrated that the ziprasidone group had a significantly lower incidence of adverse reactions compared to the olanzapine group, with particular advantages in weight gain, somnolence, and abnormal metabolic parameters. For patients requiring long‐term maintenance treatment for mania, drug tolerability and metabolic safety directly impact medication adherence and long‐term prognosis. Therefore, due to its lower risk of metabolic syndrome, ziprasidone may be more suitable for patients with concerns such as hyperglycemia, dyslipidemia, or weight control, especially among adolescents, women, or those with cardiometabolic comorbidities (Findling et al. 2013).

This study also included a comparative analysis of biological markers such as BDNF and T_3_. The results indicated that the ziprasidone group showed greater improvement in BDNF levels compared to the olanzapine group, suggesting that it may not only be effective in symptom control but could also exert therapeutic effects by enhancing neuroplasticity and neuroprotective mechanisms. BDNF is a critical factor regulating neuronal growth, synaptic plasticity, and emotional states, and its levels are closely associated with the development and progression of BD (Inal‐Emiroglu et al. 2015). On the other hand, ziprasidone had a lesser impact on T_3_ levels compared to olanzapine, indicating a lower potential for endocrine disruption, which is particularly relevant for patients requiring monitoring of thyroid function.

Although this study integrates existing research evidence through a systematic approach, offering strong comprehensiveness and clinical value, it still has the following limitations. First, all included literature originates from mainland China, which may introduce regional and publication bias. Although funnel plots did not indicate significant bias, regional limitations may affect the generalizability of the results internationally. Second, most studies exhibit methodological flaws in design and reporting, particularly in insufficient description of randomization details, allocation concealment, and blinding implementation, which may introduce certain selection and detection biases. Third, the types of mood stabilizers used across the included studies were not entirely consistent (e.g., lithium carbonate or sodium valproate), and some studies did not strictly control dosage and treatment duration, potentially affecting the pooled analysis results. Additionally, follow‐up periods were generally short, making it difficult to evaluate long‐term efficacy and relapse rates.

Based on the above findings, ziprasidone combined with mood stabilizers showed certain advantages in reducing YMRS scores and safety in the treatment of acute mania, while its overall clinical efficacy was similar to that of olanzapine combination therapy. However, this is not entirely consistent with the results of Huang et al. (2025). Besides the limitations mentioned previously, potential reasons for this discrepancy may include the following: (1) Their study involved an international multicenter population, whereas the studies included in our analysis were primarily retrieved from specific Chinese and English databases, with more concentrated study designs and population structures. This could lead to population differences (e.g., proportion of mixed episodes, baseline severity) affecting effect estimates. (2) Their study incorporated various “SGA + lithium/valproate” combinations into the same network, whereas we specifically compared only “ziprasidone + mood stabilizer” versus “olanzapine + mood stabilizer”. The distribution of stabilizer types, doses, and treatment durations within these two specific intervention arms might differ. (3) Their study uniformly used Mean Difference (MD) and presented comprehensive comparisons via a league table. In addition to continuous outcomes (YMRS changes), we also pooled dichotomous outcomes such as “overall clinical response rate”. Differences in outcome measurement approaches can introduce biases of varying directions and magnitudes. (4) The high heterogeneity (*I^2^* = 77%) in our combined analysis of YMRS scores suggests that source variations might have amplified the uncertainty of the effect size.

Future research should focus on the following directions: first, designing more rigorous, multicenter, large‐sample, long‐term follow‐up RCTs to enhance the quality of evidence and external generalizability; second, standardizing outcome measures by combining objective biomarkers (such as BDNF, inflammatory factors, EEG/neuroimaging indicators) with subjective symptom assessments to improve the scientific validity and reproducibility of findings; third, exploring individualized treatment strategies to identify which patient subtypes or comorbidities are more suitable for ziprasidone or olanzapine, thereby providing a foundation for precision psychopharmacotherapy.

In conclusion, this meta‐analysis indicates that ziprasidone combined with mood stabilizers is non‐inferior to olanzapine in treating manic episodes of BD, with certain advantages in safety, metabolic impact, and improvement in biomarkers. It therefore holds potential for clinical application, particularly for long‐term treatment and populations with high metabolic risks. Further high‐quality studies are needed to validate its efficacy mechanisms and target populations to better inform its place in therapy.

## Author Contributions

Conception and design: Peiling Yao and Pingan Ni. Data analysis and interpretation: Liqin Yin. All the authors contributed to the acquisition of data, collection and assembly of data, manuscript writing, and final approval of manuscript.

## Funding

The authors have nothing to report.

## Ethics Statement

As this study involves the summary and analysis of other studies, it does not involve medical ethics approval or patient‐informed consent.

## Consent

The authors have nothing to report.

## Conflicts of Interest

The authors declare no conflicts of interest.

## Data Availability

The authors have nothing to report.

## References

[brb371139-bib-0001] Chen, X. E. 2021. “Efficacy of Ziprasidone Combined With Sodium Valproate in the Treatment of Bipolar Disorder Manic Episodes.” Guide of China Medicine 19, no. 29: 46–48.

[brb371139-bib-0002] Chen, Z. X. , Z. B. Ren , D. S. Qiu , et al. 2014. “Ziprasidone Combined With Mood Stabilizers in the Treatment of Bipolar Manic Episodes.” Chinese Journal of General Practice 12, no. 5: 682–685.

[brb371139-bib-0003] Cichoń, L. , M. Janas‐Kozik , A. Siwiec , et al. 2020. “Clinical Picture and Treatment of Bipolar Affective Disorder in Children and Adolescents.” Psychiatria Polska 54, no. 1: 35–50. 32447355 10.12740/PP/OnlineFirst/92740

[brb371139-bib-0004] Citrome, L. , C. Graham , A. Simmons , et al. 2021. “An Evidence‐Based Review of OLZ/SAM for Treatment of Adults With Schizophrenia or Bipolar I Disorder.” Neuropsychiatric Disease and Treatment 17: 2885–2904. 34526769 10.2147/NDT.S313840 PMC8437420

[brb371139-bib-0005] Findling, R. L. , I. Cavuş , E. Pappadopulos , et al. 2013. “Efficacy, Long‐Term Safety, and Tolerability of Ziprasidone in Children and Adolescents With Bipolar Disorder.” Journal of Child and Adolescent Psychopharmacology 23, no. 8: 545–557. 10.1089/cap.2012.0029. 24111980 PMC3804078

[brb371139-bib-0006] Fredskild, M. U. , S. Stanislaus , K. Coello , et al. 2021. “Impact of Modification to DSM‐5 Criterion A for Hypomania/Mania in Newly Diagnosed Bipolar Patients: Findings From the Prospective BIO Study.” International Journal of Bipolar Disorders 9, no. 1: 14. 33937949 10.1186/s40345-020-00219-9 PMC8089066

[brb371139-bib-0007] Gao, J. , Y. Huang , and M. Li . 2019. “Does Antagonism at 5‐HT2A Receptors Potentiate D_2_ Blockade‐Induced Disruption of Conditioned Avoidance Response?” Experimental and Clinical Psychopharmacology 27, no. 2: 103–108. 30556732 10.1037/pha0000243

[brb371139-bib-0008] Gao, Y. S. , Y. H. Chen , L. J. Liang , et al. 2019. “Effects of Ziprasidone Combined With Sodium Valproate on Serum T3 and BDNF Levels in Patients With Bipolar Disorder Manic Episodes.” Journal of Guangdong Pharmaceutical University 35, no. 1: 137–139.

[brb371139-bib-0009] Haddad, H. W. , E. Boardman , B. Williams , et al. 2022. “Combination Olanzapine and Samidorphan for the Management of Schizophrenia and Bipolar 1 Disorder in Adults: A Narrative Review.” Health Psychology Research 10, no. 3: 34224. 35774916 10.52965/001c.34224 PMC9239397

[brb371139-bib-0010] Hu, L. R. , Z. Y. Ji , and Y. Q. Shao . 2019. “Analysis of the Efficacy of Different Dual‐Drug Regimens in Patients With Bipolar Disorder Manic Episodes.” Modern Practical Medicine 31, no. 9: 1275–1277.

[brb371139-bib-0011] Huang, W. , S. He , M. Liu , et al. 2025. “Comparative Efficacy, Safety, and Tolerability of Pharmacotherapies for Acute Mania in Adults: A Systematic Review and Network Meta‐Analysis of Randomized Controlled Trials.” Molecular Psychiatry 30, no. 3: 838–847. 39191865 10.1038/s41380-024-02705-3

[brb371139-bib-0012] Inal‐Emiroglu, F. N. , N. Karabay , H. Resmi , et al. 2015. “Correlations Between Amygdala Volumes and Serum Levels of BDNF and NGF as a Neurobiological Markerin Adolescents With Bipolar Disorder.” Journal of Affective Disorders 182: 50–56. 25973783 10.1016/j.jad.2015.04.016

[brb371139-bib-0013] Jing, Y. , E. Kim , M. You , A. Pikalov , and Q. V. Tran . 2009. “Healthcare Costs Associated With Treatment of Bipolar Disorder Using a Mood Stabilizer Plus Adjunctive Aripiprazole, Quetiapine, Risperidone, Olanzapine or Ziprasidone.” Journal of Medical Economy 12, no. 2: 104–113. 10.3111/13696990903044092. 19527195

[brb371139-bib-0014] Kishi, T. , T. Ikuta , Y. Matsuda , et al. 2021. “Mood Stabilizers and/or Antipsychotics for Bipolar Disorder in the Maintenance Phase: A Systematic Review and Network Meta‐Analysis of Randomized Controlled Trials.” Molecular Psychiatry 26, no. 8: 4146–4157. 33177610 10.1038/s41380-020-00946-6 PMC8550938

[brb371139-bib-0015] Luo, J. Z. , Z. L. Sun , and J. L. Chen . 2012. “Efficacy of Ziprasidone Combined With Sodium Valproate in the Treatment of Bipolar Disorder Manic Episodes.” Chinese Remedies & Clinics 12, no. 5: 668–669.

[brb371139-bib-0016] Okpaleke Amazu, C. , K. Nathwani , P. M. Berwerth , et al. 2024. “Managing a Complex Case of Bipolar Disorder in a Patient With Recurrent Hospitalizations.” Cureus 16, no. 7: e64271. 39130846 10.7759/cureus.64271 PMC11315583

[brb371139-bib-0017] Page, M. J. , J. E. Mckenzie , P. M. Bossuyt , et al. 2021. “The PRISMA 2020 Statement: An Updated Guideline for Reporting Systematic Reviews.” Bmj 372: n71. 33782057 10.1136/bmj.n71 PMC8005924

[brb371139-bib-0018] Raeder, R. , M. Arora , M. Bertocci , et al. 2025. “Reward Magnitude‐Specific Delay Discounting Differentiates Mania Versus Depression Risk.” Cognitive, Affective & Behavioral Neuroscience 25, no. 5: 1–12. 10.3758/s13415-025-01307-y PMC12577757 40437312

[brb371139-bib-0019] Salazar De Pablo, G. , D. Guinart , B. A. Cornblatt , et al. 2020. “Demographic and Clinical Characteristics, Including Subsyndromal Symptoms Across Bipolar‐Spectrum Disorders in Adolescents.” Journal of Child and Adolescent Psychopharmacology 30, no. 4: 222–234. 32083495 10.1089/cap.2019.0138 PMC7232658

[brb371139-bib-0020] Stahl, S. M. , and D. K Shayegan . 2003. “The Psychopharmacology of Ziprasidone: Receptor‐Binding Properties and Real‐World Psychiatric Practice.” Journal of Clinical Psychiatry 64, no. S19: 6–12. 14728084

[brb371139-bib-0021] Sun, Y. J. 2021. “Efficacy of Sodium Valproate Sustained‐Release Tablets Combined With Ziprasidone Hydrochloride in the Treatment of Bipolar Affective Disorder Manic Episodes.” Modern Diagnosis and Treatment 32, no. 7: 1048–1049.

[brb371139-bib-0022] Tiwari, A. K. , D. Zhang , J. G. Pouget , et al. 2018. “Impact of Histamine Receptors H1 and H3 Polymorphisms on Antipsychotic‐Induced Weight Gain.” World Journal of Biological Psychiatry 19, no. S3: S97–S105. 10.1080/15622975.2016.1262061. 27855565

[brb371139-bib-0023] Turan, S. , Ç. Ermiş , Ş. Eray , et al. 2023. “Psychomotor Agitation and Irritability in Adolescents With Manic Episode: Clinical Data From Three Inpatient Units.” Clinical Child Psychology and Psychiatry 28, no. 4: 1266–1278. 36052859 10.1177/13591045221125331

[brb371139-bib-0024] Wang, X. L. , H. J. Ma , H. T. Zou , et al. 2018. “Clinical Efficacy of Ziprasidone Combined With Sodium Valproate in the Treatment of Bipolar Disorder Manic Episodes.” Journal of International Psychiatry 45, no. 1: 62–64.

[brb371139-bib-0025] Wen, R. H. , and S. Liu . 2014. “Therapeutic Effect of Ziprasidone Combined With Sodium Valproate on Bipolar Disorder Manic Patients With Poor Response to Sodium Valproate Monotherapy.” China Modern Medicine 21, no. 34: 83–85.

[brb371139-bib-0026] Xu, Z. Y. 2022. “Comparison of the Efficacy of Ziprasidone Versus Olanzapine Combined With Sodium Valproate in the Treatment of Bipolar Disorder Manic Episodes.” World Journal of Complex Medicine 8, no. 1: 138–141.

[brb371139-bib-0027] Yang, S. S. , and X. L. Liu . 2018. “A Comparative Study on the Efficacy and Safety of Ziprasidone Sequential Therapy Versus Olanzapine in the Treatment of Bipolar Disorder With Mixed Episodes.” Jilin Medical Journal 39, no. 12: 2290–2291.

[brb371139-bib-0028] Zhou, R. , M. He , J. Fan , et al. 2022. “The Role of Hypothalamic Endoplasmic Reticulum Stress in Schizophrenia and Antipsychotic‐Induced Weight Gain: A Narrative Review.” Frontiers in Neuroscience 16: 947295. 10.3389/fnins.2022.947295. 36188456 PMC9523121

